# *Staphylococcus aureus* interaction with *Pseudomonas aeruginosa* biofilm enhances tobramycin resistance

**DOI:** 10.1038/s41522-017-0035-0

**Published:** 2017-10-19

**Authors:** T. Beaudoin, Y. C. W. Yau, P. J. Stapleton, Y. Gong, P. W. Wang, D. S. Guttman, V. Waters

**Affiliations:** 1 Translational Medicine, Research Institute, Hospital for Sick Children, 555 University Avenue, Toronto, M5G 1X8 Canada; 2 Division of Microbiology, Department of Pediatric Laboratory Medicine, The Hospital for Sick Children, University of Toronto, 555 University Avenue, Toronto, M5G 1X8 Canada; 3 Centre for the Analysis of Genome Evolution and Function, University of Toronto, Ontario, Canada; 4 Department of Cell and Systems Biology, University of Toronto, Toronto, Ontario Canada; 5 Division of Infectious Diseases, Department of Pediatrics, The Hospital for Sick Children, University of Toronto, 555 University Avenue, Toronto, M5G 1X8 Canada

## Abstract

Antimicrobial resistance is a significant threat to the treatment of infectious disease. Multiple mechanisms of resistance to different classes of antibiotics have been identified and well-studied. However, these mechanisms are studied with bacteria in isolation, whereas often, infections have a polymicrobial basis. Using a biofilm slide chamber model, we visualized the formation and development of clinical *Pseudomonas aeruginosa* biofilms in the presence of secreted *Staphylococcus aureus* exoproducts, two bacteria that commonly co-infect pediatric patients with cystic fibrosis. We showed that, over time, certain isolates of *P. aeruginosa* can form different biofilm architecture in the presence of *S. aureus* exoproducts. We further determined that this interaction was dependent on Psl produced by *P. aeruginosa* and staphylococcal protein A from *S. aureus*. Importantly, we identified a mechanism of antibiotic resistance to tobramycin that is dependent on the polymicrobial interactions between these two bacteria. This interaction occurred in isolates of *P. aeruginosa* recovered from children with cystic fibrosis who failed to clear *P. aeruginosa* following inhaled tobramycin treatment.

## Co-infection: interactions that increase resistance

The interaction between two different bacterial species can enhance the antibiotic resistance of biofilms. Resistance to antimicrobial therapy is usually studied using individual bacterial species, but many infections involve more than one species. Researchers in Canada, led by Trevor Beaudoin at The Hospital for Sick Children in Toronto, studied the effect of products secreted by *Staphylococcus aureus* bacteria on biofilms of *Pseudomonas aeruginosa*. They worked with biofilms that had resisted treatment, isolated from patients with cystic fibrosis. The co-infection altered the biofilm structure and increased its resistance to the antibiotic tobramycin. The effect depended on bacterial aggregation stimulated by the interaction between a large carbohydrate from *P. aeruginosa* and a protein secreted by *S. aureus*. The research highlights the need to study bacterial interactions to understand and treat infections involving different bacteria.

## Introduction

Antimicrobial resistance presents a major challenge in the effective treatment of infectious diseases, resulting in increased patient morbidity and mortality.^1^ The challenge of antimicrobial resistance is further complicated by the fact that results of traditional, planktonic susceptibility testing of single, bacterial species often do not correlate with clinical outcomes.^2^ It is increasingly being recognized that many infections, such as pulmonary infections occurring in patients with cystic fibrosis (CF), are polymicrobial in nature, characterized by the growth of organisms within communities known as biofilms.^3–5^

Most children with CF are initially colonized with *Staphylococcus aureus* in their airways. As these children age, *S. aureus* is most commonly replaced with *Pseudomonas aeruginosa*, often time resulting in a period of co-infection, suggesting that there may be an interaction between these organisms.^6,7^ Upon initial acquisition of *P. aeruginosa*, CF patients are treated with antibiotics in an attempt to eradicate the organism to prevent chronic colonization and the associated long-term adverse outcomes.^8^ Despite the high intrapulmonary concentrations achievable with inhaled tobramycin, *P. aeruginosa* eradication fails in 10–40% of CF patients.^9^ Studies have not identified host characteristics or consistent *P. aeruginosa* factors that can predict failure of eradication treatment.^10–12^ However, there have been few investigations into how bacterial interactions may contribute to the inability to clear *P. aeruginosa* from the lungs of CF patients.

The goal of this study was to determine how *S. aureus* exoproducts affect the establishment and persistence of *P. aeruginosa* biofilms using a unique collection of new onset *P. aeruginosa* isolates obtained from CF children before undergoing standardized Antibiotic Eradication Therapy.^12,13^ By using *S. aureus* filtrates rather than co-culturing *S. aureus* and *P. aeruginosa* in a simple competition assay, we have identified an interaction between the secreted *S. aureus* product, staphylococcal protein A (SpA), and Psl, a *P. aeruginosa* exopolysaccharide, leading to aggregation and increased tobramycin resistance in *P. aeruginosa* isolates obtained from patients who have failed eradication therapy.

## Results

### Effect of *S. aureus* exoproducts on *P. aeruginosa* in a biofilm slide chamber model

New onset *P. aeruginosa* isolates were collected from 46 patients with cystic fibrosis undergoing standardized Antibiotic Eradication Therapy with inhaled tobramycin.^12,13^ Thirty-three *P. aeruginosa* isolates were obtained from patients who underwent successful eradication therapy (eradicated isolates) and 13 isolates obtained from patients who failed eradication therapy (persistent isolates).

**Fig. 1:**
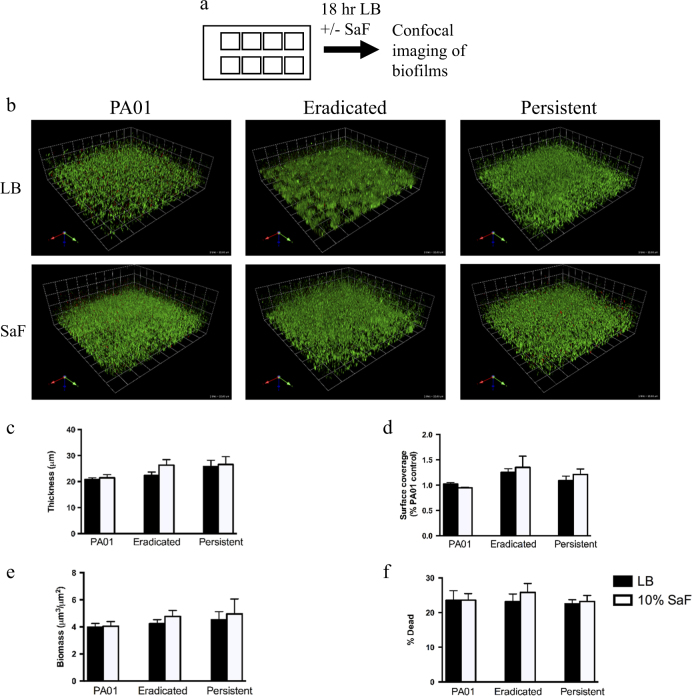
*Staphylococcus aureus* filtrates (SaF) do not affect initial attachment *of Pseudomonas aeruginosa* to borosilicate slide chambers. **a** Schematic representation of how SaF biofilm formation was assessed. *P. aeruginosa* was grown overnight in liquid culture prior to seeding in 8-well slide chambers. After 6 h of attachment, media was removed and replaced with fresh media. **b** Representative images of *P. aeruginosa* biofilms grown for 24 h in the presence of 10% (v/v) SaF in LB. Green cells: live; red cells: dead. **c**–**f** Image analysis of biofilm thickness **c**, surface coverage **d**, biomass **e** and % dead cells **f** for given biofilms. Biofilms were grown as described above in the presence of LB alone (black bars) or 10%SaF (white bars) prior to image acquisition and analysis with COMSTAT. *N* = 7 eradicated isolates and *N* = 7 persistent isolates. The mean of PA01 was generated from seven biological replicates. All means are plotted with standard deviation. Statistics were performed using non-parametric one-way ANOVA (Kruskal–Wallis) with Dunn’s post-test for multiple comparisons

To assess the effect of *S. aureus* on *P. aeruginosa*, we exposed *P. aeruginosa* isolates to *S. aureus* filtrates (SaF) (bacteria-free supernatants of *S. aureus*). We selected seven eradicated and seven persistent isolates that had similar phenotypes (with regards to mucoid status, protease production, swimming, and twitching motility, attachment in a crystal violet assay (Supplemental Table 1) and minimum inhibitory concentration (MIC) (Supplemental Table 2) in order to examine other bacterial factors influencing the success of eradication therapy. These isolates were studied using a static slide chamber model with confocal microscopic visualization. In conjunction with image analysis using Volocity and COMSTAT software, (http://www.comstat.dk/).^14,15^ this model allowed biofilm architecture to be quantitatively assessed. The presence of SaF did not affect planktonic growth (Supplemental Fig. 1) or swimming or twitching motility or protease production (Supplemental Fig. 2). The effect of SaF on initial *P. aeruginosa* biofilm formation is shown in Fig. 1. The presence of SaF had no effect on initial biofilm formation in this model system. There was no change in biofilm thickness (average height of biofilm-Fig. 1c), surface coverage (surface area of biofilm exposed on substratum and edges-Fig. 1d), biomass (biovolume per unit surface area-Fig. 1e) or % dead (ratio of dead signal to total signal-Fig. 1f) between untreated and SaF treated *P. aeruginosa* biofilms.

**Fig. 2:**
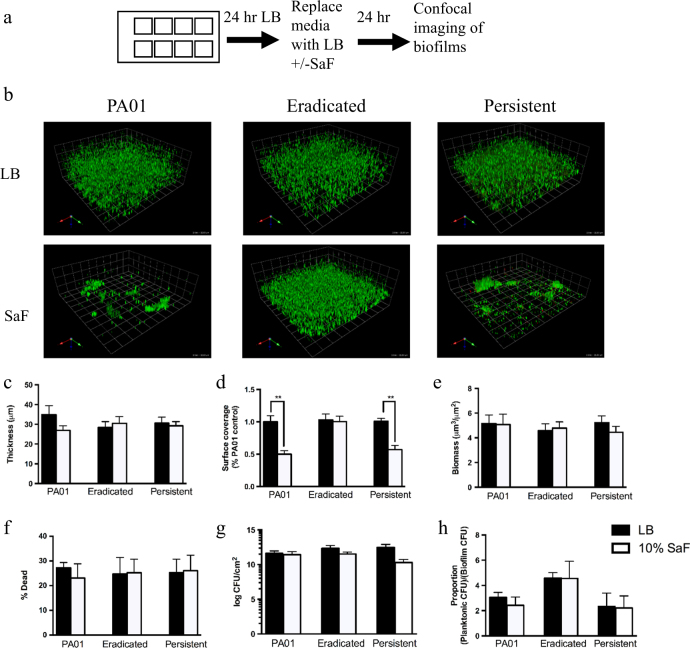
*Staphylococcus aureus* filtrates (SaF) differentially affects *Pseudomonas aeruginosa* development in a slide chamber model. **a** Schematic representation of how SaF biofilm formation was assessed. **b** Representative images of *P. aeruginosa* biofilms grown for 24 h in the presence of 10% (v/v) SaF in LB. Green cells: live; red cells: dead. **c**–**f** Image analysis of biofilm thickness **c**, surface coverage **d**, biomass **e** and % dead cells **f** for PAO1, eradicated (*N* = 7) and persistent (*N* = 7) *P. aeruginosa* isolates biofilms. Biofilms were grown as described above in the presence of LB alone (black bars) or 10%SaF (white bars) prior to image acquisition and analysis with COMSTAT. The mean of PA01 was generated from seven biological replicates. **g** CFU/cm^2^ of biofilms grown with or without SaF. **h** Proportion of CFU of unattached bacteria (in media) to attached bacteria (on surface) in LB or 10% SaF. All means are plotted with standard deviation. Statistics were performed using non-parametric one-way ANOVA (Kruskal–Wallis) with Dunn’s post-test for multiple comparisons. **p < 0.001

To determine the effect of SaF on formed biofilms, we allowed *P. aeruginosa* to grow in media alone prior to the addition of SaF for 24 h (Fig. 2a–h). When SaF was added to preformed (24-h growth) biofilms, persistent isolates had significantly reduced surface coverage in the presence of SaF compared to the untreated condition (Fig. 2d). However, biofilm thickness (Fig. 2c), biomass (Fig. 2e), % dead (Fig. 2f) was unchanged. Additionally, colony forming unit (CFU) counts of biofilms grown in SaF were not different between the treated (SaF) and untreated conditions (Fig. 2g). Furthermore, the proportion of cells in the planktonic and biofilm fractions of wells was similar in the SaF treated and untreated conditions. In contrast, eradicated isolates were not affected by SaF treatment in this model (Fig. 2h). Similar results can be seen if *P. aeruginosa* is grown in the continuous presence of SaF for 48 h (Supplemental Fig. 3). Taken together, these results indicate that SaF caused densely packed cellular aggregation evident in the reduction of the surface coverage of *P. aeruginosa*, without affecting the biomass or the viability of persistent isolates; this effect was not observed in eradicated isolates. *S. aureus* filtrate from an additional three clinical isolates and the ATCC Oxford *S. aureus* were tested against PAO1 and the persistent isolate; all showed a similar ability to form aggregates (Supplemental Fig. 4).

These aggregates were seen with the addition of SaF to preformed persistent isolate biofilms, even if the biofilms were only exposed for a short duration (e.g., 3 or 6 h-Supplemental Fig. 5a). Alternatively, if biofilms were formed for 24 h in the presence of SaF and then exposed to fresh LB alone, little aggregation was visualized (Supplemental Fig. 5b). This suggested that the aggregative effects of SaF seen in this model were dependent on having a formed biofilm.

### Aggregation caused by *S. aureus* exoproducts leads to tobramycin resistance in persistent isolates of *P. aeruginosa*

**Fig. 3:**
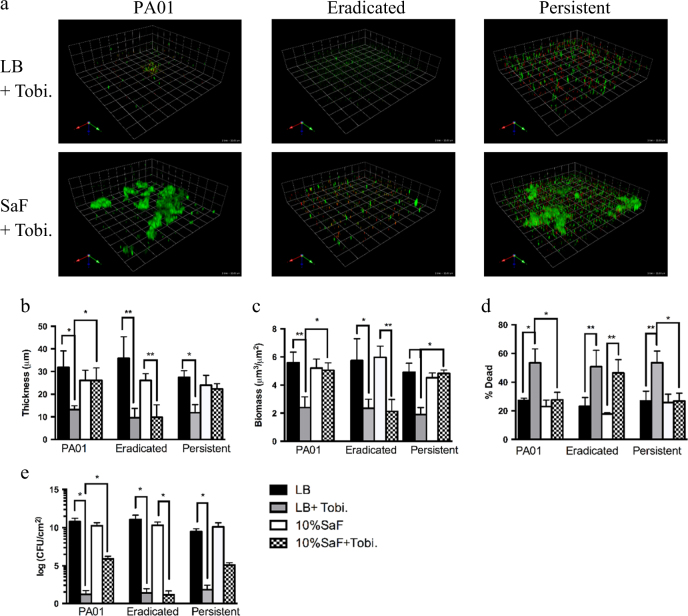
*Staphylococcus aureus* filtrates (SaF) leads to increased tobramycin resistance in *P. aeruginosa* biofilms formed from persistent isolates A static biofilm slide chamber method was used to asses the effect of *S. aureus* filtrates (SaF) on PAO1, eradicated (*N* = 7) and persistent (*N* = 7) *P. aeruginosa* antibiotic tolerance. **a** Representative images of *P. aeruginosa* biofilms grown for 24 h in LB followed by exposure to 10% SaF and or 1000 µg/mL of tobramycin for an additional 24 h prior to staining. Green cells: live; red cells: dead. **b–e** Image analysis of biofilm thickness **b**, biomass **c** % dead cells **d** and CFU counts/cm^2^ **e** for given biofilms. Biofilms were grown as described above in the presence of LB alone (black bars), LB + tobramycin (gray bars), 10%SaF (white bars) or 10%SaF + tobramycin (checkered bars) prior to image acquisition and analysis with COMSTAT. Each biological replicate consisted of analyzing six images per isolate. The mean of PA01 was generated from seven biological replicates. All means are plotted with standard deviation. Statistics were performed using non-parametric one-way ANOVA (Kruskal–Wallis) with Dunn’s post-test for multiple comparisons. **p < 0.001, *p < 0.05

To understand the clinical observation of failure of eradication therapy with inhaled tobramycin, we tested whether the presence of SaF resulted in increased resistance to tobramycin (at concentrations achievable with aerosolization).^16^ We grew *P. aeruginosa* biofilms for 24 h. Following 24 h of growth, media was removed and replaced with media containing 1000 µg/mL of tobramycin, with and without SaF, for an additional 24 h (Fig. 3a). As seen in Fig. 3, all isolates could be killed by tobramycin when grown in media alone. There was decreased biofilm thickness (Fig. 3b) and biomass (Fig. 3c) and an increase in percent of biofilm that was dead (Fig. 3d) for all isolates when grown in media alone and treated with tobramycin. Likewise, eradicated isolates grown in the presence of 10% SaF and exposed to tobramycin behaved similarly. However, there was no statistically significant difference between tobramycin-treated and untreated conditions with respect to biofilm thickness, biomass or % dead for persistent isolates or PAO1 (Fig. 3b, c, d respectively) when grown in SaF and exposed to tobramycin. Importantly, the presence of tobramycin in LB alone resulted in a significant decrease in biofilm biomass and an increase in % dead when compared to the SaF + Tobi condition for PAO1 and persistent isolates, suggesting an increased resistance to tobramycin in the presence of SaF for these isolates; this was not seen in the eradicated isolates. This antimicrobial resistance was also reflected in the increased number of viable bacterial cells measured by CFU for PAO1 and all persistent isolates when grown in SaF and tobramycin; this effect was not seen with eradicated isolates (Fig. 3e). The presence of SaF did not change the planktonic MIC of the tested isolates (Supplemental Table 2).

### Identification of *S. aureus* exoproduct causing *P. aeruginosa* biofilm aggregation

**Fig. 4:**
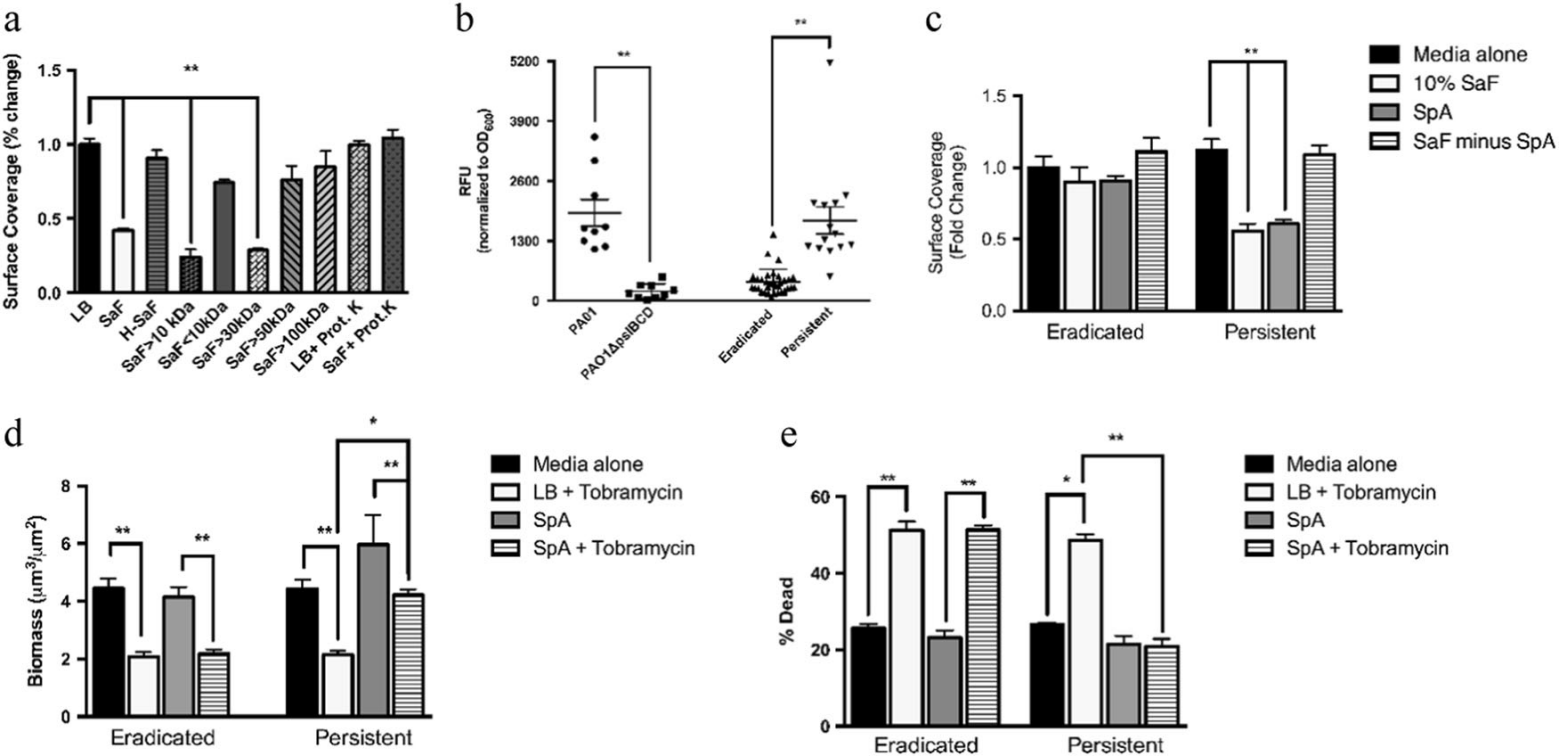
Staphylococcal protein A leads to aggregation in *Pseudomonas aeruginosa* biofilms and increases antibiotic tolerance. **a** Persistent isolate (PA580) grown in LB for 24 h followed by 24 h of growth in 10% SaF that had undergone various manipulations and analyzed for % surface coverage as a marker for aggregation. H-SaF; Heat-inactivated SaF, Prot. K: Proteinase K (0.5 mg/mL). Images from *n* = 3 independent experiments were analyzed. Mean values are plotted with standard deviation **b** FITC-SpA assay of *Pseudomonas aeruginosa* isolates grown for 24 h in 96-well microtiter plate in LB media alone. After 24 h of growth, cells were spun down and suspended in 50 µg/mL of FITC-labeled SpA. For eradicated (*n* = 33) and persistent isolate (*n* = 13), each dot represents the mean for an individual isolate obtained from 2 biological replicates. For each biological replicate, all isolates were performed in quadruplicate. For PAO1 (*n* = 9) and PAO1Δ*pslBCD* (*n* = 9), each dot represents a biological replicate. Mean values are plotted with standard deviation. **c** Eradicated or persistent isolates were grown for 24 h in LB followed by 24 h in LB + /− 50 µg/mL SpA or SaF with SpA removed via IgG sepharose column. *N* = 3 eradicated or persistent isolates performed in 3 independent experiments. Isolates were grown in LB alone (black bars), 10% SaF (white bars), 50 µg/mL SpA (dark gray bars) or SaF with SpA removed (hashed bars). Images were analyzed with COMSTAT and mean surface coverage is shown. **d**–**e** Eradicated isolates or persistent isolates were grown for 24 h in LB followed by 24 h in LB + /− 50 µg/mL SpA with or without tobramycin. *N* = 3 eradicated or persistent isolates performed in 3 independent experiments. Average biomass **d** and % dead **e** of biofilms as described above in LB (Black bars), LB + tobramycin (White bars), SpA (dark gray bars) or SpA + tobramycin *(hatched bars*). All means are plotted with standard deviation. Statistics were performed using non-parametric one-way ANOVA (Kruskal–Wallis) with Dunn’s post-test for multiple comparisons. **p < 0.001, *p < 0.05

To determine what could be causing this specific interaction leading to aggregation, we performed several manipulations on the SaF (Fig. 4a). Using a series of molecular weight cut-off filters to create fractions which excluded materials of different sizes, we determined that the active product was a heat labile protein that was between 30 and 50 kDa in size. In light of a recent study^17^ that identified SpA as a binding partner to *P. aeruginosa*, we focused on this protein to determine whether it could generate the phenomenon observed using SaF. Using a SpA ELISA, we determined its concentration in the filtrate material to be between 1–14 µg/mL (Supplemental Table 3). Additionally, we could effectively remove SpA from our filtrates by using IgG sepharose columns and confirmed this using the SpA ELISA and gel electrophoresis (not shown).

The ability of SpA to bind to eradicated and persistent isolates from our cohort was determined using a fluorescently labeled SpA conjugate (SpA-FITC) in a 96-well plate assay. As seen in Fig. 4b and Supplemental Table 4, most eradicated isolates had lower SpA binding than did the persistent isolates. The mean relative fluorescence units (RFU) for PAO1 was 1312 RFU, while for the PA01Δ*pslBCD* deletion isolate it was 220RFU (*p* < 0.001, Kruskal–Wallis test). For the entire cohort of eradicated isolates (*n* = 33)), the mean RFU was 477 while for persistent isolates (*n* = 13) the mean RFU was 1472 (*p* < 0.001, Kruskal–Wallis test). One persistent isolate (PA565) had very high SpA binding, but even when this isolate was removed from analysis, the mean RFU of SpA binding in the persistent group was significantly higher than that of the eradicated group. When grown in a slide chamber for 48 h, the presence of SpA could reduce the surface coverage of biofilm formed by persistent isolates, similar to the effects that were seen with SaF (Fig. 4c). Additionally, passing filtrate through IgG sepharose columns to remove SpA, abrogated the effects of the SaF (Fig. 4c). Furthermore, the addition of purified SpA could protect persistent isolates from tobramycin, resulting in greater biomass (Fig. 4d) and less % dead (Fig. 4e) compared to biofilms formed without SpA present. Eradicated isolates were unaffected by the addition of SpA (Fig. 4d–e).

To determine whether SaF induced a change at the transcriptomic level of *P. aeruginosa*, 24-h biofilms were exposed to SaF for 3 h and RNA was isolated and processed using RNASeq. There were no statistically significant differences in gene expression between the treated and untreated conditions (Supplemental Fig. 6).

**Fig. 5:**
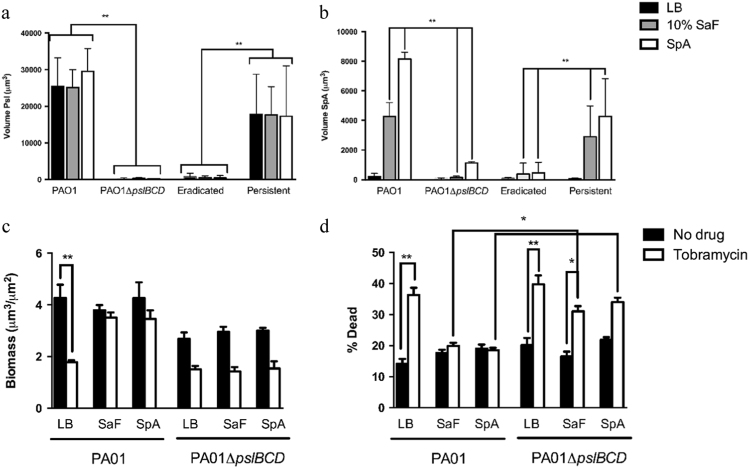
Binding of Staphylococcal protein A to Psl exopolysaccharide in* Pseudomonas aeruginosa* biofilms leads to increased tobramycin resistance. **a–b** Total volume of Psl stain **a** and SpA **b** in biofilm as determined by voxel counts using Volocity software. *N* = 4 eradicated and *N* = 4 persistent isolates performed *n* = 3 times. Mean values are plotted with standard deviation. **c–d** Biofilms were grown for 48 h in the presence of or absence of SaF or SpA. Following the initial 24 h period, media was removed and replaced with fresh media with or without SaF or Spa and with or without tobramycin. Images were taken and analyzed for average biomass **c** and % dead **d** of biofilms as described. Black bars represent biofilms without tobramycin, white bars represent biofilms grown in presence of tobramycin. Experiment was performed *n* = 3 times. Mean values are plotted with standard error of the mean. Statistics were performed using non-parametric one-way ANOVA (Kruskal–Wallis) with Dunn’s post-test for multiple comparisons. **p < 0.001, *p < 0.05 compared to LB control

Therefore, we looked for a potential interaction between SpA and *P. aeruginosa*.Given that SpA has been shown to interact with Psl,^17^ we examined how eradicated and persistent isolates behaved in the presence of SpA using a Psl stain and labeled SpA antibody. As seen in Fig. 5, there was no Psl staining and little SpA binding in eradicated isolates, quantified as the volume of Psl (Fig. 5a) and SpA (Fig. 5b). Conversely, persistent isolates showed strong anti-SpA antibody staining after exposure to SaF or purified SpA. In these conditions, Psl and SpA staining co-localized, with 70–95% signal overlap seen within the formed aggregates. Using wild-type PAO1 and a Psl deletion strain (PAO1Δ*pslBCD*) we showed that *P. aeruginosa* strains lacking Psl cannot bind SpA efficiently (Fig. 5b). Representative images of these experiments can be found in Supplemental Fig. 7. To determine whether the observed interaction was associated with antimicrobial resistance, we examined the ability of tobramycin (our standard antibiotic treatment for eradication) to kill *P. aeruginosa* isolates in the presence of SpA. Using PAO1 and the Psl deletion strain (PAO1Δ*pslBCD*) we showed that the Psl negative isolate was not protected from tobramycin killing in the presence of SaF or SpA (Fig. 5c–d). The addition of SpA to PAO1 resulted in a greater biomass and decreased % dead in the presence of tobramycin compared to the LB control. For the PAO1ΔpslBCD isolate, there was a trend towards decreased biomass and increased killing with tobramycin, under the three conditions (LB alone, with SaF or SpA, Fig. 5c, d).

### Aggregation caused by SpA-Psl binding leads to tobramycin resistance in persistent isolates of *P. aeruginosa*

**Fig. 6:**
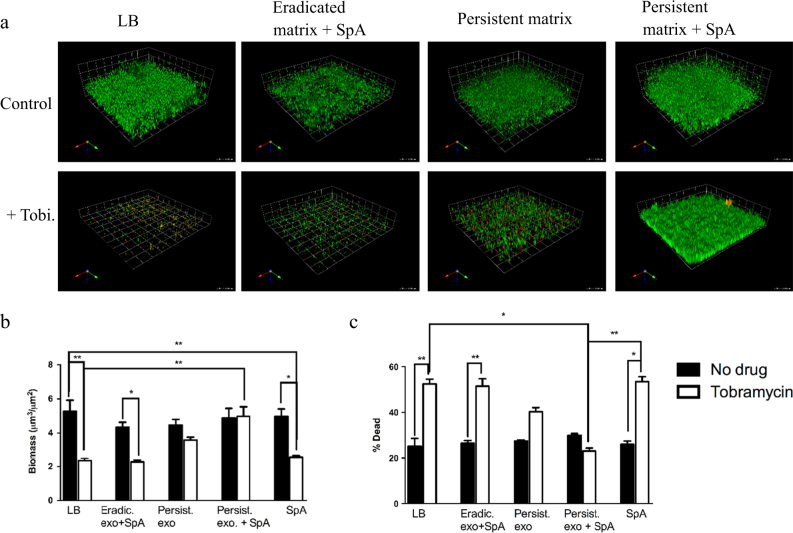
Exogenous exopolysaccharides derived from PA01 or persistent isolates, and in the presence of SpA, can protect eradicated isolates biofilms from tobramycin. **a** Representative images of eradicated isolate grown in the presence of exopolysaccharides produced from different isolates of *Pseudomonas aeruginosa*. Eradicated isolates were grown for 24 h in media alone, followed by 24 h in the presence of 10% extracted biofilm matrix from an eradicated isolate with 50 µg/mL SpA (Eradic. Exo + SpA) or from biofilm matrix obtained from a persistent isolate in the presence or absence of 50 µg/mL SpA for 2 h. Following 2 h treatment, tobramycin was added to a final concentration of 1000 µg/mL and biofilm was allowed to grow for an additional 24 h before imaging. Images were obtained and analyzed for, biomass **b** and % dead **c** of biofilms without (Black bars) tobramycin or in the presence (White bars) of tobramycin. *N* = 3 Eradicated isolates performed *n* = 3 times. Mean values are plotted with standard deviation. Statistics were performed using non-parametric one-way ANOVA (Kruskal–Wallis) with Dunn’s post-test for multiple comparisons. **p < 0.001, *p < 0.05 compared to LB control

To determine whether the matrix from PAO1 or persistent isolates could confer protection in eradicated isolates, the crude matrix from persistent isolates was added to eradicated isolates (Fig. 6). Crude matrix from persistent isolates alone provided a small level of protection to eradicated *P. aeruginosa* isolates following exposure to tobramycin, as observed in Fig. 6. However, the addition of this extract along with SpA, provided protection to tobramycin killing in eradicated isolates similar to what was seen in persistent isolates (Fig. 6a–c). The crude matrix obtained from eradicated isolates did not confer any protection to tobramycin killing, even in the presence of SpA (Fig. 6a–c).

## Discussion

In this study, we investigated the effects of *S. aureus* exoproducts on the ability of *P. aeruginosa* to form biofilms, in the context of cystic fibrosis, and demonstrated that interspecies bacterial interactions can induce changes in biofilm architecture in a time-dependent manner. We identified the novel finding that *P. aeruginosa* Psl binding to SpA induces bacterial aggregation leading to tobramycin resistance in isolates that have persisted despite tobramycin eradication treatment.

Secreted factors such as SpA have previously been shown to interact with different host factors, however, it has only recently been described to impact bacterial–bacterial interactions. A study by Armbruster et al.^17^ noted an interaction between SpA and *P. aeruginosa* Psl. In the absence of Psl, components of *S. aureus* filtrates, such as SpA, were shown to inhibit biofilm formation as measured by crystal violet assays.^17^ They attributed this lack of attachment to binding of SpA to type 4 pili in *P. aeruginosa* when Psl was not present, resulting in decreased attachment. In our study, however, there was no significant impediment to initial biofilm growth amongst any of the isolates studied when *P. aeruginosa* was grown on a chambered coverglass. This difference in observations was likely attributable to the fact that biofilms grown on coverslips rely on initial flagella attachment whereas attachment in crystal violet assays is more dependent on type 4 pili interaction with the surface.^18,19^ The advantage of our current model was the ability to directly visualize the effect of these interactions once a biofilm was established.

In our model, once biofilm was formed, in the continuous presence of *S. aureus* exoproducts, *P. aeruginosa* formed dense aggregates of bacteria on the surface of coverslips. These changes in biofilm structure only occurred after an initial period of biofilm growth by *P. aeruginosa* and only in wild-type PA01 and persistent isolates. Furthermore, these changes appeared to be dependent on SpA-Psl binding. Psl is a charge-neutral polysaccharide made up of repeating pentasaccharide units and has been well described for its role in cell–cell and cell–substrate attachment adhesion and biofilm formation in vitro.^20–28^ It has been previously reported that Psl production in *P. aeruginosa* biofilm is under temporal and spatial regulation.^20^ Overhage et al. described the role of Psl in the initial attachment of the biofilm monolayers, followed by repression of Psl; as the biofilm further develops, Psl is again expressed specifically in micro-colonies. Psl expression in the periphery of biofilms has also been shown to influence the development of micro and macro-colonies of *P. aeruginosa* biofilms.^26^ Taken together, these results and our data identify the importance of temporal regulation of Psl expression in the structure and architecture of the biofilm extracellular matrix.

Additionally, Psl has been implicated in providing generic resistance to certain antimicrobials.^28^ In this study, Billings et al. using mixed-species biofilms to show that *S. aureus* was protected from tobramycin killing when grown with *P. aeruginosa* capable of producing Psl, suggesting it provides a generalized protection against antimicrobial killing. We confirmed this finding and additionally showed that the presence of *S. aureus* components, like SpA, can enhance the antimicrobial resistance of *P. aeruginosa* biofilms via aggregate formation. Our results highlight the additional role of interspecies interactions in increasing tobramycin resistance in *P. aeruginosa* biofilms.

It is important to note that not all *P. aeruginosa* isolates behaved the same in the presence of *S. aureus* exoproducts. While none of the eradicated isolates tested showed impairment in forming a biofilm in our model, they did seem impaired in their ability to form aggregates, particularly in the presence of *S. aureus* filtrates or SpA, whereas the persistent isolates (and PAO1) readily formed aggregates. The results using our model suggested that there was a difference in Psl between the eradicated and persistent isolates. The exact nature of the difference in Psl between eradicated and persistent isolates is currently unknown. Although there was much weaker binding of the lectin stain (a specific marker for mannose that distinguishes between Psl, Pel, and alginate exopolysaccharide in *P. aeruginosa*) in the eradicated compared to persistent isolates, it is not clear why this occurred. There were no deletions in the Psl operon or promoter region in these isolates and initial experiments demonstrated that most of the eradicated isolates tested did produce detectable Psl (data not shown). Whether there are differences in Psl quantity, composition or location (i.e., cell associated vs. secreted) that contribute to our observed phenomenon, is unknown. Without this information, it is difficult to speculate why SpA leads to increased aggregation in our model. Whether it is active recruitment of *P. aeruginosa* to sites containing SpA or an increased binding of *P. aeruginosa* through a physical interaction with SpA and Psl, is unknown. Interestingly, secreted SpA has been associated with biofilm formation and *S. aureus* aggregation previously.^29^ The accumulation of SpA in PAO1 and Persistent isolates may led to a similar phenomenon.

The present study has several limitations. Firstly, the use of an abiotic surface and rich media source like lysogeny broth does not reflect the in vivo conditions faced by *P. aeruginosa* in the CF lung. However, the strength of this model system is that it allows for visualization of biofilm architecture and the changes that occur to biofilms with bacterial interactions, which cannot be assessed in standard crystal violet assays. Another limitation is the focus on the interaction between two organisms present in the CF lung in isolation of host factors. It is well recognized that there are many factors, both from the host^30–32^ and other microorganisms present, which can affect *P. aeruginosa* persistence. The aggregation observed with SpA-Psl binding may be reflective of a more generalized phenotype of *P. aeruginosa*. There are many possible triggers of *P. aeruginosa* auto-aggregation including cleavage of flagella by neutrophil elastase and secretion of the cdrA adhesion, among others.^31,33^ Given the retrospective nature of this study, there was no remaining sample of the original sputum from patients with eradicated and persistent isolates to examine for *S. aureus* co-infection using molecular methods or measure sputum SpA concentrations. We, therefore, cannot say with certainty what role SpA-Psl interactions may have played in eradication failure in these patients. Finally, the total number of isolates used in this study was relatively small and the findings require validation in a secondary collection of new onset *P. aeruginosa* isolates, such as those collected from subjects in the Early Pseudomonas Infection Control interventional trial.

There are undoubtedly many factors that contribute to the outcome of initial antimicrobial treatment of *P. aeruginosa*. However, the distinction between eradicated and persistent isolates based on SpA-Psl binding in our Eradication Study Cohort suggests that differences in Psl, either in amount or composition, for example, may exist and be clinically relevant in the management of early *P. aeruginosa* infection in CF. Currently, there is no way of predicting who will successfully eradicate initial *P. aeruginosa* infection. Children with CF who fail the first attempts at eradication may undergo further antibiotic treatment steps. However, the success of each additional antibiotic treatment subsequently decreases and longer time to the initiation of therapy diminishes the probability of a successful outcome.^13^ Understanding how Psl differs between eradicated and persistent isolate would not only allow clinicians to pre-emptively identify those at risk of failure of eradication therapy, but also identify new targets for more effective alternative treatments.

In summary, the work described herein highlights the importance of bacterial interactions in the context of antimicrobial resistance. We have shown that secreted products from *S. aureus* can interact with *P. aeruginosa* Psl to enhance the aggregate formation and induce antibiotic resistance in clinical *P. aeruginosa* isolates that have failed to be eradicated by high-dose tobramycin treatment in children with CF. Additional research is needed to identify the characteristics of Psl associated with failure of eradication therapy in order to improve the management of early *P. aeruginosa* infection in CF.

## Methods

### Bacterial strains

Clinical isolates were obtained from a cohort of pediatric CF patients undergoing eradication treatment with inhaled tobramycin for new-onset *P. aeruginosa* infection at the Hospital for Sick Children (Toronto) from 2011–2014.^12^ Since 2010, children with CF with new onset *P. aeruginosa* have undergone a standardized Antibiotic Eradication Therapy protocol with 28 days of inhaled tobramycin treatment followed by a repeat respiratory tract culture 1 week later (off antibiotics); if the repeat culture was positive, they received subsequent treatments steps as previously outlined. The initial *P. aeruginosa* isolates (before antibiotic treatment) were selected from seven patients in whom *P. aeruginosa* was successfully eradicated with inhaled tobramycin (eradicated strains) and from seven patients in whom eradication therapy failed (persistent strains).

*S. aureus* isolates were cultured from sputum specimens collected from four pediatric CF patients followed at the Hospital for Sick Children who never cultured positive for *P. aeruginosa*. All bacterial strains were stored at −80 °C in glycerol.

### Filtrate production

Single colonies of *S. aureus* were grown overnight in 3 mL of lysogeny broth (LB, Lennox formulation) in 14 mL loosely capped test tubes. Twenty microliter of this overnight culture was then diluted into 3 mL of fresh LB and grown for an additional 48 h. Following growth, cells were separated from excreted products by centrifugation (3000×*g*, 30 min) and filter sterilized through a 0.22 μm low-binding filter and pooled. One milliliter from each batch of filtrates was saved and underwent a compatibility treatment to be used for BCA testing to get the total protein content of the filtrates. Sterility of filtrates was assured by plating 100 μL of filtrates onto a blood agar plate.

### Biofilm growth on chamber slides

*P. aeruginosa* was grown in chamber slides as previously described for other bacteria.^34–36^ Briefly, clinical isolates of *P. aeruginosa* were grown overnight in 3 mL of lysogeny broth (Lennox formulation-LB) with shaking, overnight. Forty microliter of overnight culture was diluted into 4 mL of LB and grown to an OD 600 of 0.5–06. This was diluted 1/10 and 220 µL were used to seed the wells of an 8-chambered cover-glass slide (NuncLab-Tek II, Thermo Fisher). After 6 h of attachment, media was removed and replaced with fresh media. Biofilms were allowed to grow for further 24–48 h, replacing media every 12 h until the conclusion of the experiment.

### Treatment of biofilms in chambered coverglass

Pre-formed biofilms in chambered coverglass were also exposed to 10% SaF that had undergone various manipulations as described below. *Staphylococcus aureus* protein A was purchased from Sigma and used at a final concentration of 50 μg/mL. Biofilms treated with tobramycin (Sigma) were exposed for 24 h to 1000 µg/mL of the drug.

### Confocal microscopy

Biofilms were grown under various conditions in the chamber slide method described above. Before confocal microscopy, biofilms were stained using the Filmtracer Live/Dead biofilm viability kit (Life Technologies, Burlington, ON, Canada). The medium was gently removed from the chambers, and 200 μl total of the Live/Dead stain was added to the chambers. After 45 min of incubation, the stain was removed, and fresh medium was placed in the wells. These chamber slides were used for confocal imaging. Confocal images were acquired using a Quorum WaveFX spinning disk confocal system (Quorum Technologies Inc., Guelph, Canada). All images were acquired using a 25× water objective (total magnification, ×250) on a Zeiss AxioVert 200 M Microscope. Spectral Borealis lasers (green, 491 nm; red, 561 nm) were used for excitation. Emission filter sets of 515/40 and 624/40 were used to visualize the SYTO9 and propidium iodide stains, respectively. For each experiment, each isolate tested was performed in duplicate technical replicate (two chambers per condition) and four images per chamber were captured for analysis. Each experiment had three biological replicates (three independent experiments).

### Image analysis

Volocity software (PerkinElmer, Guelph, Canada) was used for acquisition and analysis of images. Once images were captured from the microscope they were analyzed using Volocity software to get mean fluorescence from each channel. This was used to estimate the percentage of dead cells in the biofilm by using the mean fluorescence of the (red channel/(mean red + mean green channel fluorescence))×100. The total volume of the voxels in the green channel (HHA-FITC) and red (SpA-Texas Red) for the entire image stacks were used as a parameter of Psl and SpA binding respectively. Three-dimension images were also generated using Volocity software. OME-TIFF files were then created for COMSTAT analysis (http://www.comstat.dk/).^14,15^ Additional information in Supplemental Methods.

### Staphylococcal protein A binding assay

Staphylococcal protein A binding was assessed using a 96-well microtiter assay using a FITC labeled-SpA staining procedure. *P. aeruginosa* isolates were grown overnight and subculture to an OD of 0.062 in LB media. One hundred microliter of this was then used to inoculate a 96-well black plate used for fluorescent reading. Following 24 h of growth at 37 °C, OD600 was taken and 10 µl of media was removed from each well for CFU counts. Cells were spun down at 3000×*g* for 20 min. Media was removed and wells were gently washed with 150 µL of PBS. Following this cell pellets were re-suspended 125 µL of PBS with 50 µg/mL of SpA-FITC (Sigma–Aldrich) was added to each well and plate was incubated at room temperature for 2 h in the dark. Cells were then spun down, media removed and washed 2× in PBS. The plate was then read with a fluorescent cell reader with excitation at 488 nm and emission readings at 535 nm with top and bottom read performed.

### Removal of SpA from filtrates

Thirty milliliter of SaF were prepared and ran through an IgG Sepharose column as per manufactures instructions. Pass through was tested for activity on persistent strains in the chamber slide model. Eluent from the columns was lyophilized and suspended in 10 mL of LB. This was tested for activity in the chamber slide model.

### SpA and Psl staining of Biofilms

*P. aeruginosa* biofilms were grown in the slide chamber method as described above. Following 24 h of growth, biofilms were treated with SaF, SpA or media alone and allowed to grow for an additional 24 h. Following this, media was removed, and biofilms were stained with 200 µL of 100 µg/mL of FITC-HHA stain (to label Psl-EY Laboratories via Cedarlane, Burlington, Ontario), diluted into fresh media, for 15 min, in the dark. After this, the stain was removed, and wells were washed 2× with fresh media. 200 µL of 1/1000 anti-SpA antibody conjugated to Texas Red (Abcam, ab7247, Toronto, Ontario) was added to each well for 1 h. Media was then removed, and cells washed 2× with fresh media. Biofilms were then visualized using confocal microscopy as described above.

### Extraction of exopolysaccharides from biofilm matrix

Exopolysaccharides were extracted from the biofilm matrix of PAO1, PA580 (persistent) and PA558 (eradicated) isolates in an adapted version of methods previously described.^23,37^ Following EPS extraction, biofilms pre-formed by eradicated strains were treated with the crude EPS products with or without the addition of purified SpA. After 2 h of pre-treatment, tobramycin was added to the wells at a final concentration of 1000 µg/mL for 24 h. Biofilms were then visualized using Live/Dead staining as described above.

### Statistical analysis

Comparison of continuous data within groups of *P. aeruginosa* isolates was done using the Kruskal–Wallis test with a Dunn’s multiple comparison post-test. A *P-*value of <0.05 was considered significant. All analyses were done using GraphPad Prism version 5.04.

### Data availability

Data generated and analyzed during this study are included in this published article and its Supplemental information file. Additional details available upon reasonable request.

## Electronic supplementary material

Supplemental methods

Supplemental table 1

Supplemental Table 2

Supplemental table 3

Supplemental table 4

Supplemental Figure 1

Supplemental Figure 2

Supplemental Figure 3

Supplemental Figure 4

Supplemental Figure 5

Supplemental Figure 6

Supplemental Figure 7
