# Supplementary material for: Staphylococcus aureus interaction with Pseudomonas aeruginosa biofilm enhances tobramycin resistance
Source: NPJ Biofilms Microbiomes. 2017 Oct 19;3:25. doi: 10.1038/s41522-017-0035-0 (PMC5648753; doi:10.1038/s41522-017-0035-0)
Supplement: Supplementary file 1 — Supplemental methods [file 41522_2017_35_MOESM1_ESM.docx]

**Supplemental Materials**

**Methods**

***P. aeruginosa* phenotypic characterization**

Swim motility assays were performed as previously described ^1^. In brief, a single colony from a blood agar plate was picked with a sterile toothpick and stabbed in the center of a swim agar plate containing 0.3 % agar. After incubation at 37°C overnight, the diameter of the halo in the agar was measured in millimeters (mm) and their means calculated to determine swim motility. Similarly, twitch motility was measured as previously described ^2^ . Briefly, after incubation on a twitch agar plate and staining with 0.1 % crystal violet solution, the diameter of the purple halo at the bottom of the plate was measured in mm.

Protease production was measured by using protease plates containing skim milk powder and agar. The plates were examined for clearing around the point of inoculation and the diameter of the zone of clearing measured in mm.

Mucoidy status was determined for each *P. aeruginosa* isolate by inoculation onto yeast extract mannitol (YEM) agar and examined for mucoidy by visual scoring according to a previously described grading system after incubation aerobically at 37 °C for 24–48 h ^3^.

To assess the effect of *S. aureus* on these phenotypic characteristics, media was cooled to 55^o^C in a water bath, prior to the addition of 10% SaF (v/v) and pouring of the plates.

**COMSTAT Image analysis**

Prior to analysis OME-TIFF files for each image were acquired using Volocity and converted using Bio-formats into readable files for COMSTAT. These images were then run using COMSTAT software with connected volume filtering and automatic thresholding with Otsu’s method to assess:

**Biomass**: The bio-volume of the biofilm is determined by the total voxel count (3 dimensional pixel with an X,Y,Z value in a Z-stack image) above the threshold of detection, multiplied by the height, length and width of a voxel. The bio-volume is divided by the area of the substratum to get a measure of bio-volume independent of area observed with units of µm^3^/µm^2^.

**Biofilm thickness**: The average height of the biofilm biomass across the area observed in µm.

**Surface coverage**: The total surface area of the biofilm as defined by the total area of the voxels that face the substratum or outer surface of the biofilm in units of µm^2^.

**Quantification of SpA in filtrates**

The amount of SpA in the pass through, eluent and original SaF (prior to manipulation) was quantified using a SpA ELISA as per manufactures protocol (Abcam, Cambridge, MA). Samples were diluted in a range from neat to 10^-7^. Additional, protein was run on an SDS-PAGE gel and underwent total blue protein (GelCode Blue, Thermo Scientific) staining as per manufacturer’s protocol.

**Minimum Inhibitory Concentration**

Tobramycin panel plates was used in cation-adjusted Mueller Hinton Broth (CAMHB) prepared. Bacterial inoculum was prepared by diluting 1.5 mL of a 0.5 McFarland standard suspension into 25 mL of sterile distilled water. Ten µL of diluted bacteria was added to each well of the antibiotic plates to achieve an inoculum of approximately 5 x 10^5^ colony forming units (CFU)/mL. Plates were then incubated under aerobic conditions at 37°C for 24 hours. The purity of the inoculum was checked by streaking 1uL of the positive control well onto Columbia agar plates with 5% sheep blood (Oxoid, Nepean, Canada). Minimum inhibitory concentrations (MICs) were determined by visually assessing the turbidity of wells for indication of bacterial growth after the 24-hour incubation period. MICs for each isolate were repeated in duplicates, and the higher MIC was recorded. If the MIC values were greater than two double dilutions, this was considered a major error and the experiment was repeated. MIC in the presence of 10% SaF was performed in the same way, except 10µL of SaF was added to 80µL of the antibiotic solution in each well of the plate, prior to addition of the bacterial isolate. The MIC of strain in the presence of crude extract was performed as described in methods. However, wells containing 10% crude extract (v/v) were included on the plate.

**RNASeq Experiments**

RNA libraries were sequenced on Illumina NextSeq in CAGEF. Sequence data in bcl format were converted to fastq format with Illumina bcl2fastq (v2.16.0.10) software. Sequences in fastq format were aligned to PAO1 genes with the short reads aligner novoalign (v3.02.06) (novocraft.com). Number of uniquely aligned reads to each gene were then counted with an in-house script. The reads count data were loaded in the R package edgeR (v3.8.6) (bioconductor.org/packages/release/bioc/html/edgeR.html) for differential gene expression analysis.

**Data availability**

RNA-seq data files are available through NCBI study accession SRP093377 or bioproject PRJNA353241.

**References**

References

1. Manos, J. *et al*. Virulence factor expression patterns in Pseudomonas aeruginosa strains from infants with cystic fibrosis. *Eur. J. Clin. Microbiol. Infect. Dis.* **32**, 1583-1592 (2013).

2. de la Fuente-Nunez, C. *et al*. Inhibition of bacterial biofilm formation and swarming motility by a small synthetic cationic peptide. *Antimicrob. Agents Chemother.* **56**, 2696-2704 (2012).

3. Zlosnik, J. E. *et al*. Differential mucoid exopolysaccharide production by members of the Burkholderia cepacia complex. *J. Clin. Microbiol.* **46**, 1470-1473 (2008).
