# Supplementary material for: Staphylococcus aureus interaction with Pseudomonas aeruginosa biofilm enhances tobramycin resistance
Source: NPJ Biofilms Microbiomes. 2017 Oct 19;3:25. doi: 10.1038/s41522-017-0035-0 (PMC5648753; doi:10.1038/s41522-017-0035-0)
Supplement: Supplementary file 2 — Supplemental table 1 [file 41522_2017_35_MOESM2_ESM.docx]

**Supplemental Table 1:** Strain information for eradicated (n=7) and persistent (n=7) isolates used for further analysis in this study. Mucoid status, protease production (in millimeters, mm)), swimming and twitching motility are represented. No significant difference was seen between groups based on two-tailed student t-test. *Staphylococcus aureus* co-infection status for eradicated and persistent *Pseudomonas aeruginosa* isolates. mm: millimeters; OD_550_: Optical Density at 550nm; SA: *Staphylococcus aureus;* +: culture positive; -: culture negative mo: months; Avg.: Average; NA: Non-applicable.

| Isolate |  | | Mucoid | Protease (mm) | Swim (mm) | Twitch (mm) | CV assay  (OD_550_) | SA positivity at collection | SA positivity 12 months prior |
| --- | --- | --- | --- | --- | --- | --- | --- | --- | --- |
|  | | **Eradicated Isolates** | | | | | | | |
| 50 |  | | Yes | 12 | 13 | 33 | 0.497 | + | 75% |
| 263 |  | | No | 8 | 14 | 36 | 0.492 | + | 100% |
| 288 |  | | No | 14 | 19 | 21 | 0.431 | - | 67% |
| 325 |  | | No | 16 | 19 | 29 | 0.478 | + | 100% |
| 404 |  | | Yes | 15 | 19 | 0 | 0.332 | + | 100% |
| 549 |  | | No | 18 | 15 | 21 | 0.602 | + | 100% |
| 558 |  | | No | 19 | 12 | 26 | 0.508 | + | 22% |
| Avg. |  | | NA | 14.5 | 13.1 | 23.7 | 0.477 | NA | NA |
|  | | **Persistent Isolates** | | | | | | | |
| PA342 |  | | Yes | 16 | 16 | 21 | 0.398 | + | 17% |
| PA375 |  | | No | 15 | 18 | 27 | 0.637 | + | 100% |
| PA380 |  | | No | 0 | 0 | 1 | 0.403 | - | 0% |
| PA505 |  | | Yes | 16 | 17 | 4 | 0.388 | + | 80% |
| PA551 |  | | No | 12 | 15 | 21 | 0.419 | - | 0% |
| PA565 |  | | No | 14 | 0 | 32 | 0.835 | + | 100% |
| PA580 |  | | No | 20 | 19 | 18 | 0.295 | - | 0% |
| Avg. |  | | NA | 13.7 | 12.17 | 17.6 | 0.482 | NA | NA |
