# Supplementary material for: Staphylococcus aureus interaction with Pseudomonas aeruginosa biofilm enhances tobramycin resistance
Source: NPJ Biofilms Microbiomes. 2017 Oct 19;3:25. doi: 10.1038/s41522-017-0035-0 (PMC5648753; doi:10.1038/s41522-017-0035-0)
Supplement: Supplementary file 3 — Supplemental Table 2 [file 41522_2017_35_MOESM3_ESM.docx]

**Supplemental Table 2:** Minimum inhibitory concentration (MIC) (µg/ml) for tobramycin of eradicated and persistent isolates grown planktonically with or without 10% (v/v) SaF added.

| Isolate | MIC | MIC in 10% SaF |
| --- | --- | --- |
| Eradicated Isolates | | |
| 50 | 4 | 8 |
| 263 | 2 | 2 |
| 288 | 1 | 1 |
| 325 | 124 | 124 |
| 404 | 1 | 1 |
| 549 | 1 | 1 |
| 558 | 2 | 2 |
| Persistent Isolates | | |
| PA342 | 2 | 2 |
| PA375 | 1 | 1 |
| PA380 | 1 | 2 |
| PA505 | 1 | 1 |
| PA551 | 8 | 8 |
| PA565 | 2 | 4 |
| PA580 | 4 | 4 |
