# Supplementary material for: Staphylococcus aureus interaction with Pseudomonas aeruginosa biofilm enhances tobramycin resistance
Source: NPJ Biofilms Microbiomes. 2017 Oct 19;3:25. doi: 10.1038/s41522-017-0035-0 (PMC5648753; doi:10.1038/s41522-017-0035-0)
Supplement: Supplementary file 4 — Supplemental table 3 [file 41522_2017_35_MOESM4_ESM.docx]

**Supplemental Table 3:** Quantification of Staphylococcal protein A in different preparations of *S. aureus* used in this study. Clinical SA isolates; N=4 isolates from 4 different CF patients culture negative for *P. aeruginosa.* The average is of 3 independent experiments. SA= *Staphylococcus aureus,* STDEV: standard deviation

| Condition | Average SpA (μg/mL) | Range | STDEV |
| --- | --- | --- | --- |
|  |  | (μg/mL) |  |
| Clinical SA isolates | 4.32 | 1.41-14.56 | 1.3 |
| Oxford SA | 3.11 | 1.55-5.3 | 1.94 |
| Sepharose- Pass through | 0.00641 | 0.000175- | 0.00725 |
|  |  | 0.01436 |  |
| Sepharose- Eluent | 14.8 | 9.1-22.31 | 6.6 |
