# Supplementary material for: Staphylococcus aureus interaction with Pseudomonas aeruginosa biofilm enhances tobramycin resistance
Source: NPJ Biofilms Microbiomes. 2017 Oct 19;3:25. doi: 10.1038/s41522-017-0035-0 (PMC5648753; doi:10.1038/s41522-017-0035-0)
Supplement: Supplementary file 5 — Supplemental table 4 [file 41522_2017_35_MOESM5_ESM.docx]

**Supplemental Table 4:** Mean relative fluorescent unit (RFU) value (n=8) of Staphylococcal protein A-FITC binding for eradicated and persistent isolates used in this study. STDEV: standard deviation

| Isolate | Mean RFU | STDEV |
| --- | --- | --- |
| PA01 | 1907 | 869 |
| PA14 | 175 | 152 |
| Eradicated Isolates | | |
| 6 | 285.1 | 151 |
| 26 | 511.6 | 763 |
| 39 | 300.2 | 409 |
| 50 | 327.3 | 194 |
| 130 | 293.9 | 238 |
| 250 | 312.5 | 249 |
| 263 | 179.5 | 233 |
| 282 | 1025 | 957 |
| 288 | 151.6 | 386 |
| 294 | 510.7 | 517 |
| 300 | 443.3 | 517 |
| 304 | 1447 | 1468 |
| 306 | 462.8 | 247 |
| 325 | 371.6 | 192 |
| 343 | 188.8 | 428 |
| 358 | 464.1 | 736 |
| 366 | 176.9 | 302 |
| 369 | 284.2 | 5.1 |
| 385 | 217 | 171 |
| 404 | 271 | 385 |
| 420 | 263.3 | 309 |
| 421 | 592.2 | 503 |
| 429 | 897.6 | 1065 |
| 450 | 80.86 | 101 |
| 472 | 451.8 | 416 |
| 510 | 526.7 | 190 |
| 549 | 272.2 | 189 |
| 558 | 163.8 | 266 |
| 569 | 197.9 | 202 |
| 575 | 555.2 | 503 |
| 579 | 397.7 | 366 |
| Persistent Isolates | | |
| 257 | 1458 | 458 |
| 295 | 1169 | 693 |
| 330 | 2067 | 680 |
| 336 | 1594 | 968 |
| 342 | 1318 | 735 |
| 375 | 1077 | 476 |
| 380 | 1218 | 921 |
| 505 | 2117 | 986 |
| 551 | 520 | 98 |
| 554 | 1128 | 454 |
| 565 | 5162 | 1780 |
| 573 | 2116 | 927 |
| 580 | 2280 | 638 |
| 583 | 1174 | 399 |
