# Supplementary material for: Staphylococcus aureus interaction with Pseudomonas aeruginosa biofilm enhances tobramycin resistance
Source: NPJ Biofilms Microbiomes. 2017 Oct 19;3:25. doi: 10.1038/s41522-017-0035-0 (PMC5648753; doi:10.1038/s41522-017-0035-0)
Supplement: Supplementary file 6 — Supplemental Figure 1 [file 41522_2017_35_MOESM6_ESM.pptx]

## Slide 1
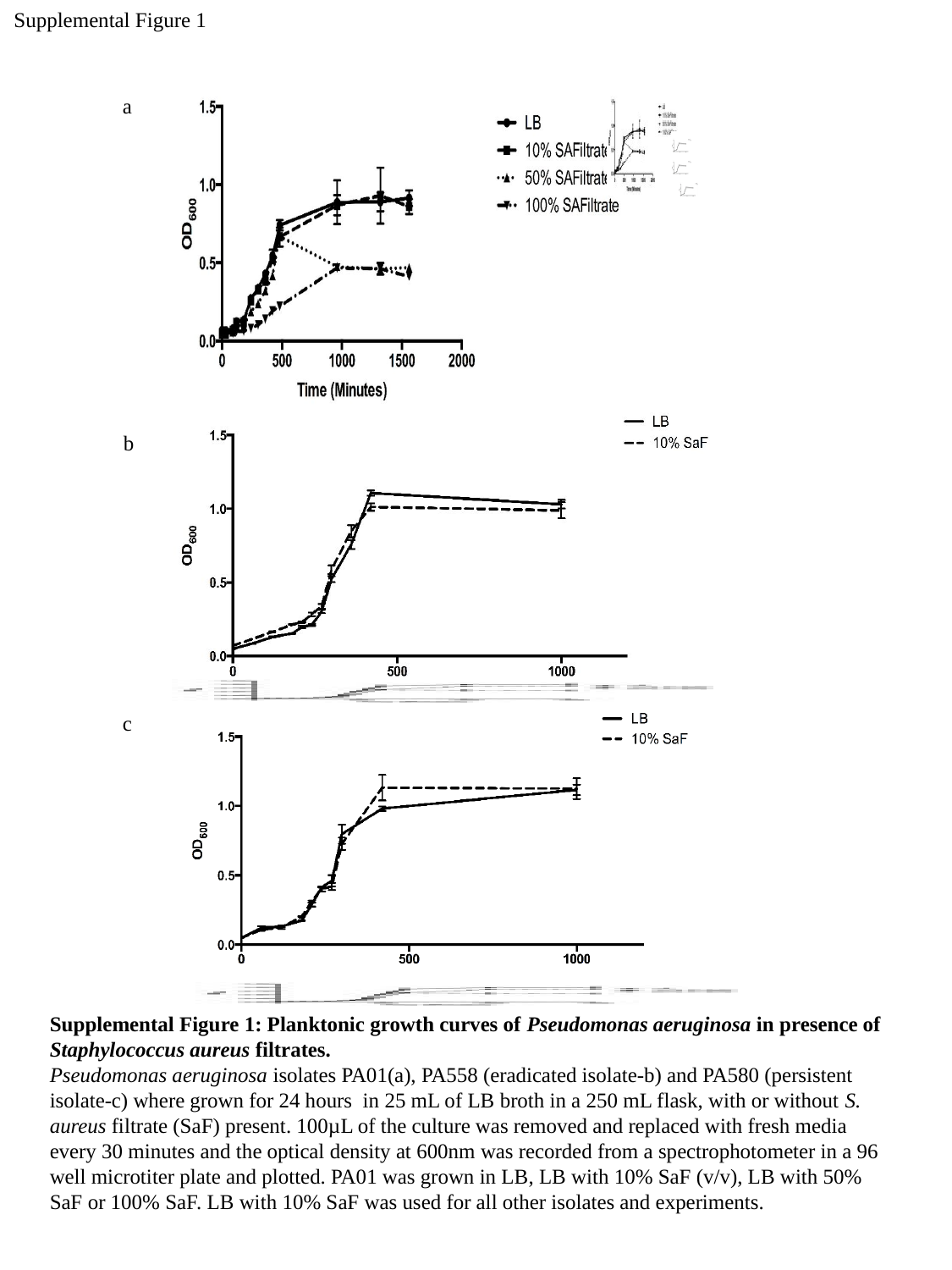

Supplemental Figure 1
a
b
c
Supplemental Figure 1: Planktonic growth curves of Pseudomonas aeruginosa in presence of Staphylococcus aureus filtrates.
Pseudomonas aeruginosa isolates PA01(a), PA558 (eradicated isolate-b) and PA580 (persistent isolate-c) where grown for 24 hours in 25 mL of LB broth in a 250 mL flask, with or without S. aureus filtrate (SaF) present. 100µL of the culture was removed and replaced with fresh media every 30 minutes and the optical density at 600nm was recorded from a spectrophotometer in a 96 well microtiter plate and plotted. PA01 was grown in LB, LB with 10% SaF (v/v), LB with 50% SaF or 100% SaF. LB with 10% SaF was used for all other isolates and experiments.
