# Supplementary material for: Staphylococcus aureus interaction with Pseudomonas aeruginosa biofilm enhances tobramycin resistance
Source: NPJ Biofilms Microbiomes. 2017 Oct 19;3:25. doi: 10.1038/s41522-017-0035-0 (PMC5648753; doi:10.1038/s41522-017-0035-0)
Supplement: Supplementary file 7 — Supplemental Figure 2 [file 41522_2017_35_MOESM7_ESM.pptx]

## Slide 1
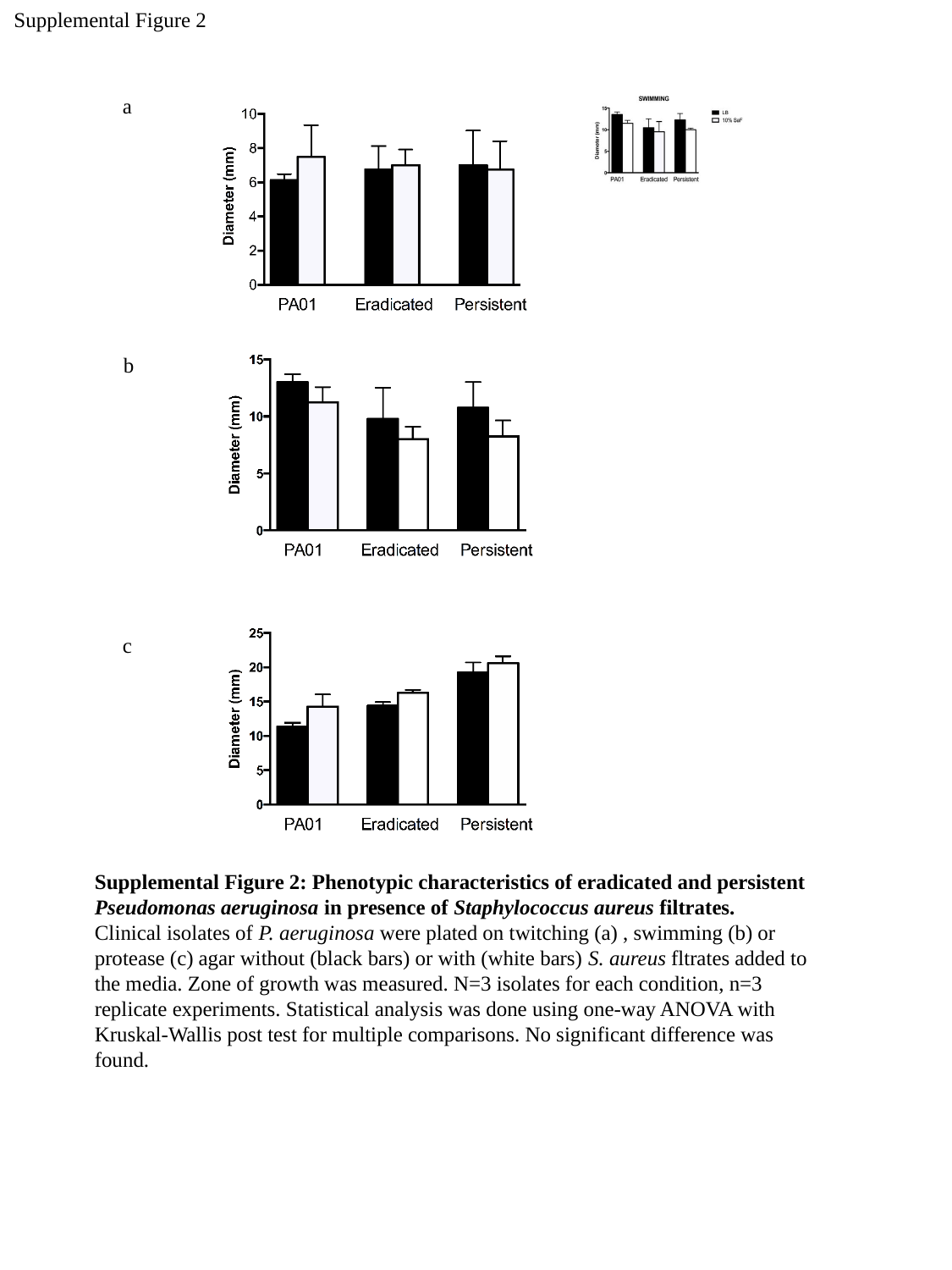

Supplemental Figure 2
a
b
c
Supplemental Figure 2: Phenotypic characteristics of eradicated and persistent Pseudomonas aeruginosa in presence of Staphylococcus aureus filtrates.
Clinical isolates of P. aeruginosa were plated on twitching (a) , swimming (b) or protease (c) agar without (black bars) or with (white bars) S. aureus fltrates added to the media. Zone of growth was measured. N=3 isolates for each condition, n=3 replicate experiments. Statistical analysis was done using one-way ANOVA with Kruskal-Wallis post test for multiple comparisons. No significant difference was found.
