# Supplementary material for: Staphylococcus aureus interaction with Pseudomonas aeruginosa biofilm enhances tobramycin resistance
Source: NPJ Biofilms Microbiomes. 2017 Oct 19;3:25. doi: 10.1038/s41522-017-0035-0 (PMC5648753; doi:10.1038/s41522-017-0035-0)
Supplement: Supplementary file 8 — Supplemental Figure 3 [file 41522_2017_35_MOESM8_ESM.pptx]

## Slide 1
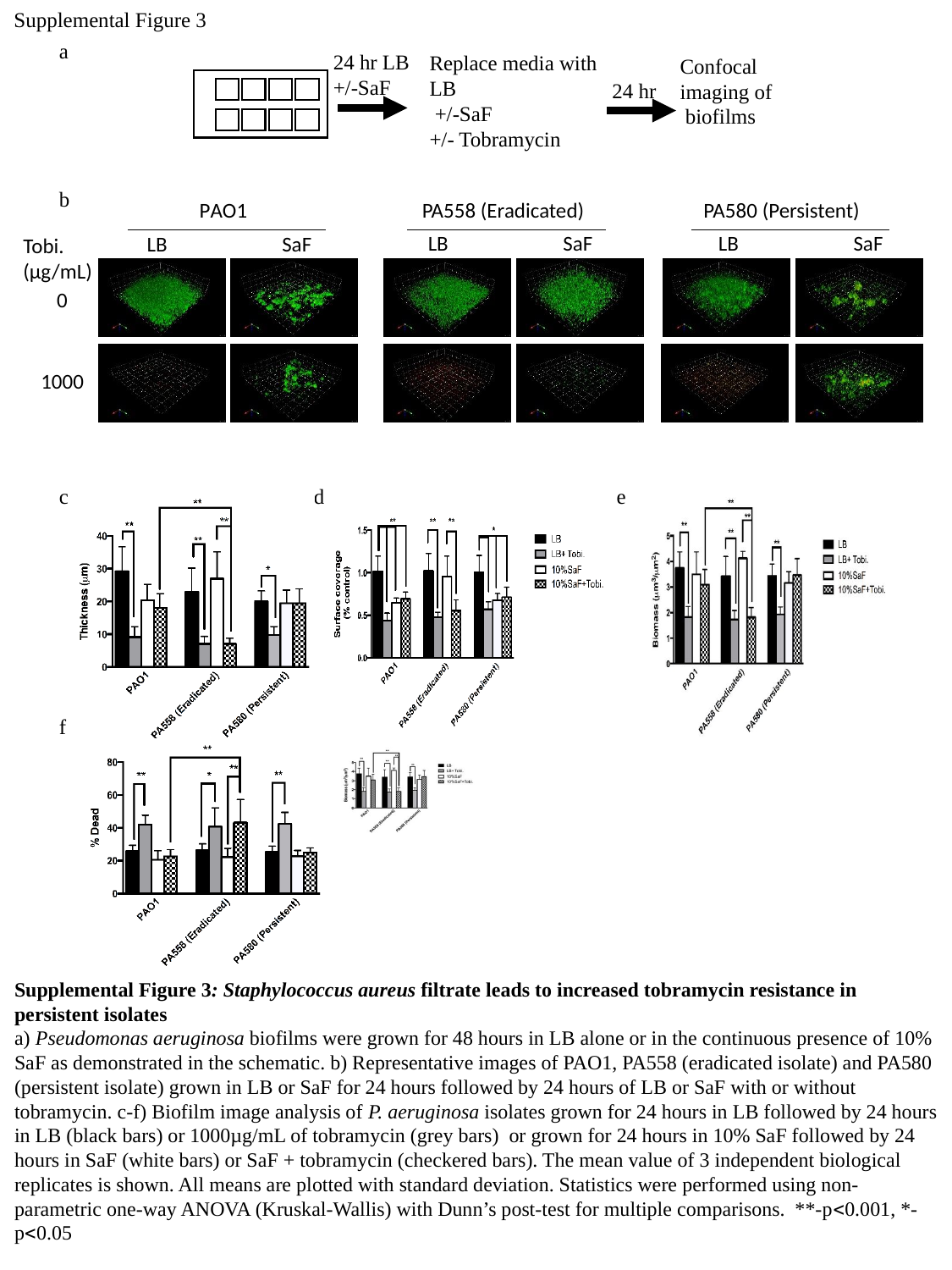

Supplemental Figure 3
a
24 hr LB
+/-SaF
Replace media with LB
 +/-SaF
+/- Tobramycin
Confocal imaging of biofilms
24 hr
b
PA558 (Eradicated)
PAO1
PA580 (Persistent)
LB
SaF
LB
SaF
LB
SaF
Tobi.
(µg/mL)
0
1000
c
d
e
f
Supplemental Figure 3: Staphylococcus aureus filtrate leads to increased tobramycin resistance in persistent isolates
a) Pseudomonas aeruginosa biofilms were grown for 48 hours in LB alone or in the continuous presence of 10% SaF as demonstrated in the schematic. b) Representative images of PAO1, PA558 (eradicated isolate) and PA580 (persistent isolate) grown in LB or SaF for 24 hours followed by 24 hours of LB or SaF with or without tobramycin. c-f) Biofilm image analysis of P. aeruginosa isolates grown for 24 hours in LB followed by 24 hours in LB (black bars) or 1000µg/mL of tobramycin (grey bars) or grown for 24 hours in 10% SaF followed by 24 hours in SaF (white bars) or SaF + tobramycin (checkered bars). The mean value of 3 independent biological replicates is shown. All means are plotted with standard deviation. Statistics were performed using non-parametric one-way ANOVA (Kruskal-Wallis) with Dunn’s post-test for multiple comparisons. **-p0.001, *-p0.05
