# Supplementary material for: Staphylococcus aureus interaction with Pseudomonas aeruginosa biofilm enhances tobramycin resistance
Source: NPJ Biofilms Microbiomes. 2017 Oct 19;3:25. doi: 10.1038/s41522-017-0035-0 (PMC5648753; doi:10.1038/s41522-017-0035-0)
Supplement: Supplementary file 9 — Supplemental Figure 4 [file 41522_2017_35_MOESM9_ESM.pptx]

## Slide 1
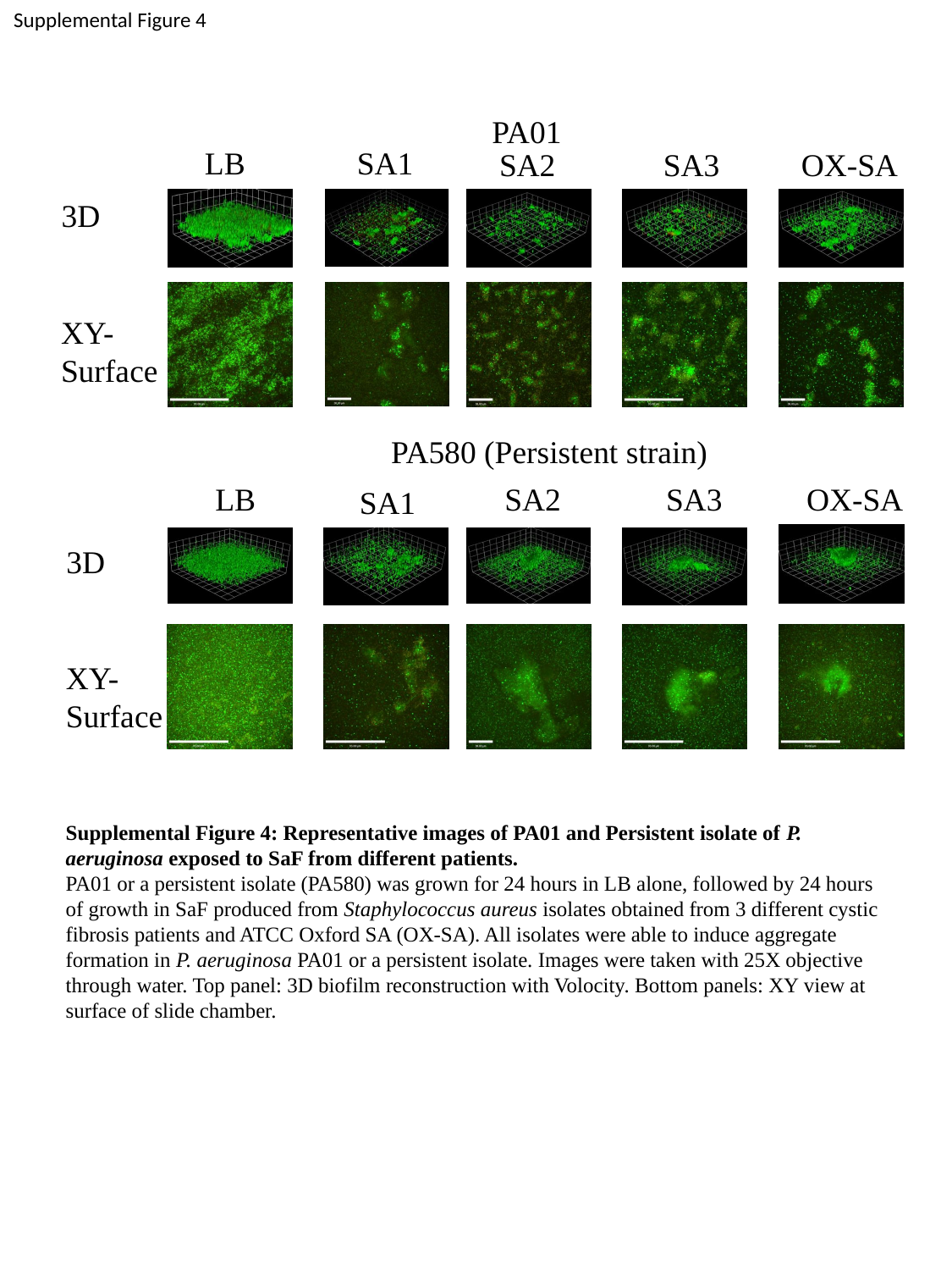

Supplemental Figure 4
PA01
LB
SA1
SA33
SA2
OX-SA
3D
XY-
Surface
PA580 (Persistent strain)
SA2
SA3
OX-SA
LB
SA1
3D
XY-
Surface
Supplemental Figure 4: Representative images of PA01 and Persistent isolate of P. aeruginosa exposed to SaF from different patients.
PA01 or a persistent isolate (PA580) was grown for 24 hours in LB alone, followed by 24 hours of growth in SaF produced from Staphylococcus aureus isolates obtained from 3 different cystic fibrosis patients and ATCC Oxford SA (OX-SA). All isolates were able to induce aggregate formation in P. aeruginosa PA01 or a persistent isolate. Images were taken with 25X objective through water. Top panel: 3D biofilm reconstruction with Volocity. Bottom panels: XY view at surface of slide chamber.
