# Supplementary material for: Staphylococcus aureus interaction with Pseudomonas aeruginosa biofilm enhances tobramycin resistance
Source: NPJ Biofilms Microbiomes. 2017 Oct 19;3:25. doi: 10.1038/s41522-017-0035-0 (PMC5648753; doi:10.1038/s41522-017-0035-0)
Supplement: Supplementary file 10 — Supplemental Figure 5 [file 41522_2017_35_MOESM10_ESM.pptx]

## Slide 1
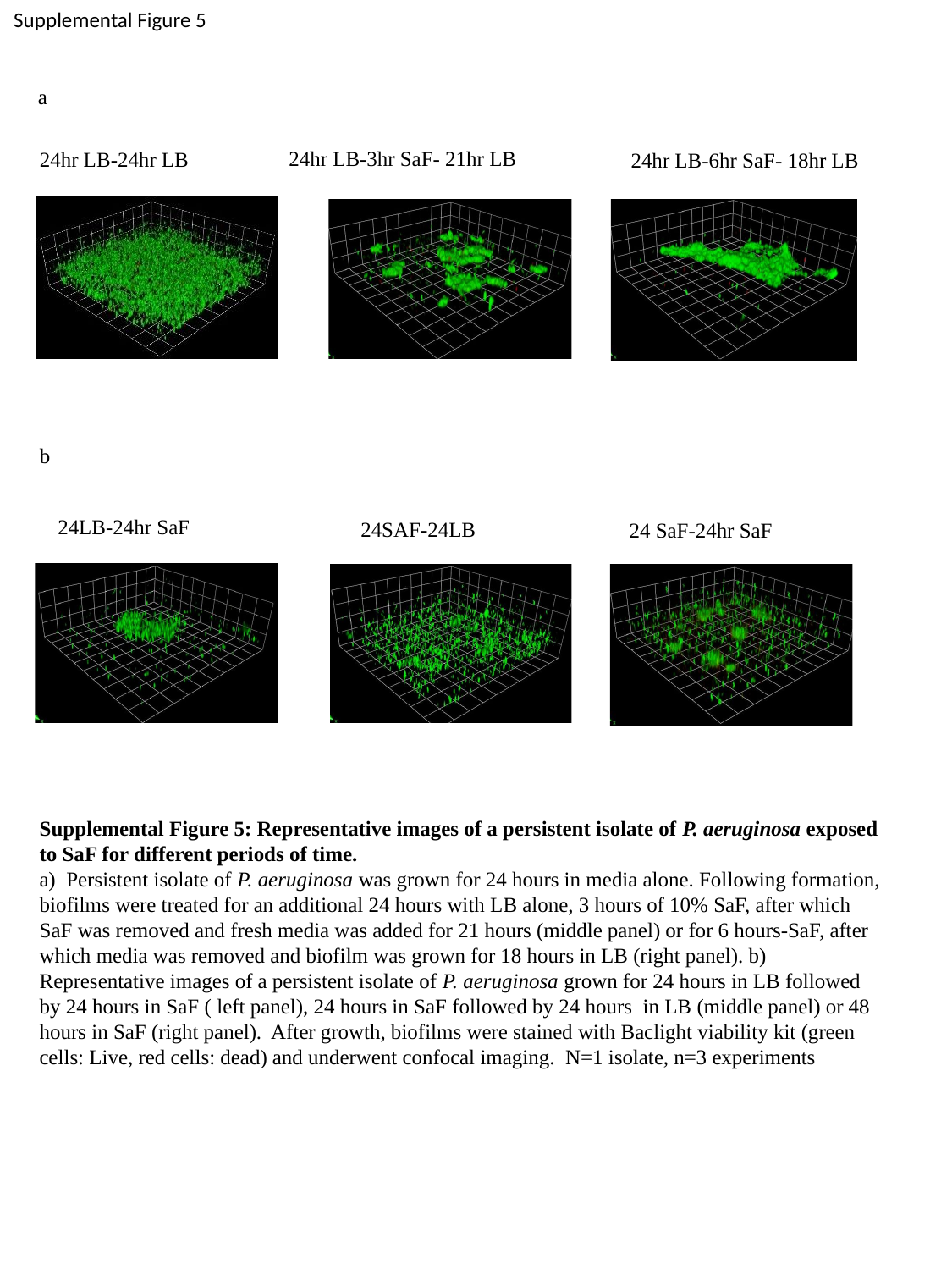

Supplemental Figure 5
a
24hr LB-3hr SaF- 21hr LB
24hr LB-24hr LB
24hr LB-6hr SaF- 18hr LB
b
24LB-24hr SaF
24SAF-24LB
24 SaF-24hr SaF
Supplemental Figure 5: Representative images of a persistent isolate of P. aeruginosa exposed to SaF for different periods of time.
a) Persistent isolate of P. aeruginosa was grown for 24 hours in media alone. Following formation, biofilms were treated for an additional 24 hours with LB alone, 3 hours of 10% SaF, after which SaF was removed and fresh media was added for 21 hours (middle panel) or for 6 hours-SaF, after which media was removed and biofilm was grown for 18 hours in LB (right panel). b) Representative images of a persistent isolate of P. aeruginosa grown for 24 hours in LB followed by 24 hours in SaF ( left panel), 24 hours in SaF followed by 24 hours in LB (middle panel) or 48 hours in SaF (right panel). After growth, biofilms were stained with Baclight viability kit (green cells: Live, red cells: dead) and underwent confocal imaging. N=1 isolate, n=3 experiments
