# Supplementary material for: Staphylococcus aureus interaction with Pseudomonas aeruginosa biofilm enhances tobramycin resistance
Source: NPJ Biofilms Microbiomes. 2017 Oct 19;3:25. doi: 10.1038/s41522-017-0035-0 (PMC5648753; doi:10.1038/s41522-017-0035-0)
Supplement: Supplementary file 11 — Supplemental Figure 6 [file 41522_2017_35_MOESM11_ESM.pptx]

## Slide 1
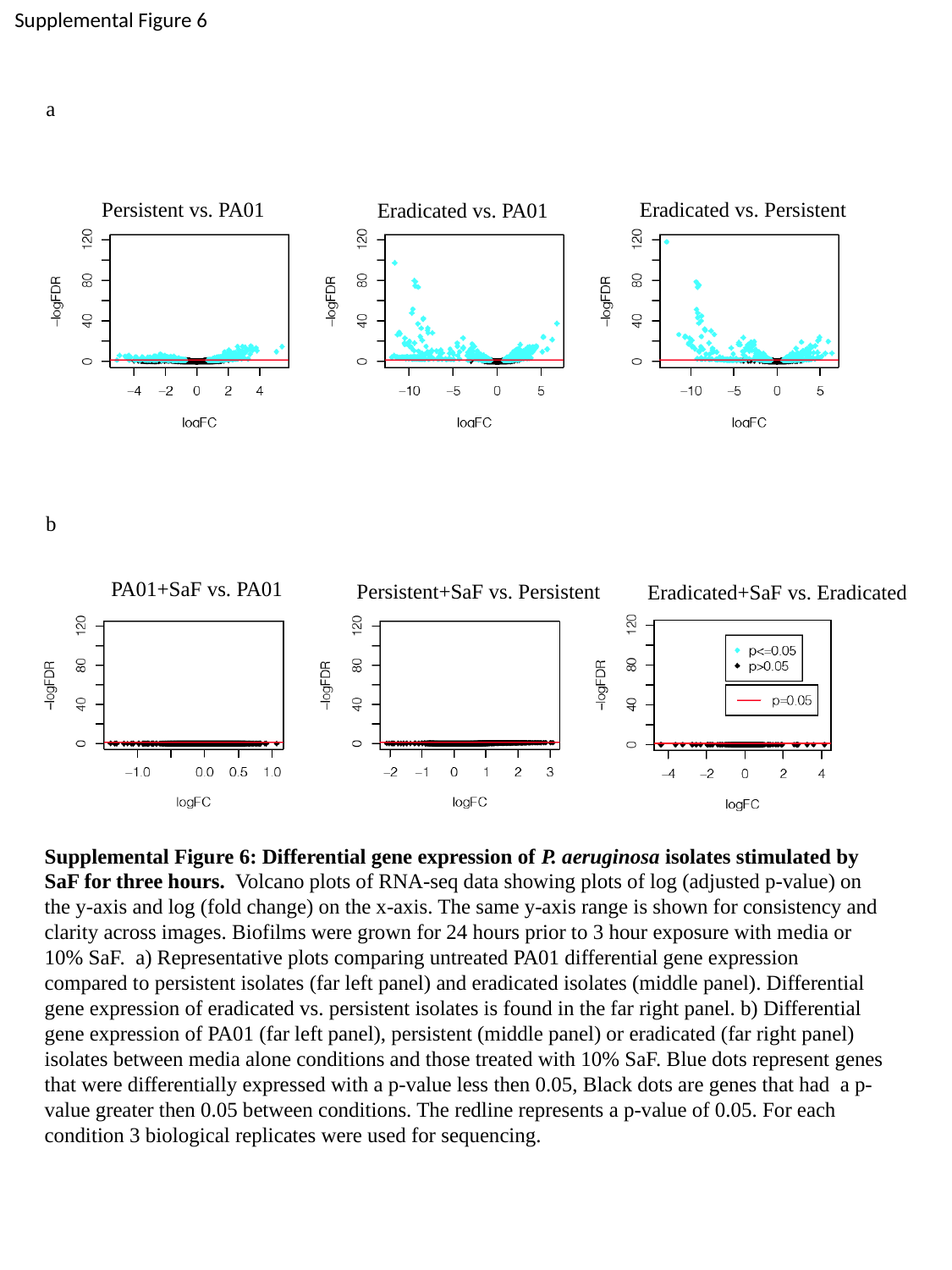

Supplemental Figure 6
a
Eradicated vs. Persistent
Persistent vs. PA01
Eradicated vs. PA01
b
PA01+SaF vs. PA01
Persistent+SaF vs. Persistent
Eradicated+SaF vs. Eradicated
Supplemental Figure 6: Differential gene expression of P. aeruginosa isolates stimulated by SaF for three hours. Volcano plots of RNA-seq data showing plots of log (adjusted p-value) on the y-axis and log (fold change) on the x-axis. The same y-axis range is shown for consistency and clarity across images. Biofilms were grown for 24 hours prior to 3 hour exposure with media or 10% SaF. a) Representative plots comparing untreated PA01 differential gene expression compared to persistent isolates (far left panel) and eradicated isolates (middle panel). Differential gene expression of eradicated vs. persistent isolates is found in the far right panel. b) Differential gene expression of PA01 (far left panel), persistent (middle panel) or eradicated (far right panel) isolates between media alone conditions and those treated with 10% SaF. Blue dots represent genes that were differentially expressed with a p-value less then 0.05, Black dots are genes that had a p-value greater then 0.05 between conditions. The redline represents a p-value of 0.05. For each condition 3 biological replicates were used for sequencing.
