# Supplementary material for: Staphylococcus aureus interaction with Pseudomonas aeruginosa biofilm enhances tobramycin resistance
Source: NPJ Biofilms Microbiomes. 2017 Oct 19;3:25. doi: 10.1038/s41522-017-0035-0 (PMC5648753; doi:10.1038/s41522-017-0035-0)
Supplement: Supplementary file 12 — Supplemental Figure 7 [file 41522_2017_35_MOESM12_ESM.pptx]

## Slide 1
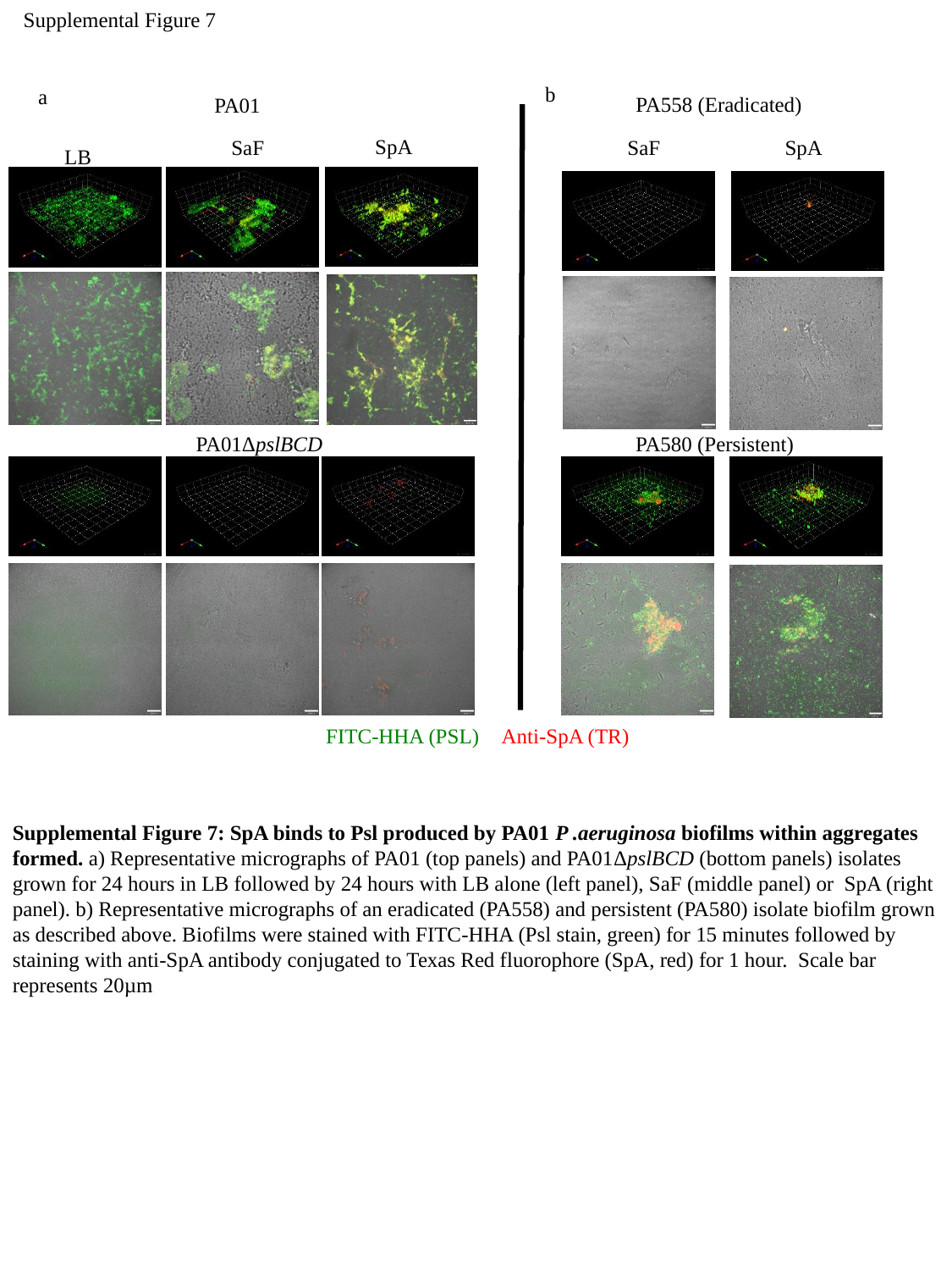

Supplemental Figure 7
b
a
PA558 (Eradicated)
PA01
SpA
SaF
SaF
SpA
LB
38μm
17μm
38μm
38μm
PA01ΔpslBCD
PA580 (Persistent)
FITC-HHA (PSL)
Anti-SpA (TR)
Supplemental Figure 7: SpA binds to Psl produced by PA01 P .aeruginosa biofilms within aggregates formed. a) Representative micrographs of PA01 (top panels) and PA01ΔpslBCD (bottom panels) isolates grown for 24 hours in LB followed by 24 hours with LB alone (left panel), SaF (middle panel) or SpA (right panel). b) Representative micrographs of an eradicated (PA558) and persistent (PA580) isolate biofilm grown as described above. Biofilms were stained with FITC-HHA (Psl stain, green) for 15 minutes followed by staining with anti-SpA antibody conjugated to Texas Red fluorophore (SpA, red) for 1 hour. Scale bar represents 20µm
